# *Thismiadomei* and *T.terengganuensis* (Thismiaceae), two new species, and *T.javanica*, a new record from Terengganu, Peninsular Malaysia

**DOI:** 10.3897/phytokeys.124.34728

**Published:** 2019-06-20

**Authors:** Siti-Munirah Mat Yunoh, Dome Nikong

**Affiliations:** 1 Forest Research Institute Malaysia, 52109 Kepong, Selangor, Malaysia Forest Research Institute Malaysia Kepong Malaysia; 2 DigitalDome Photography, 21500 Permaisuri, Terengganu, Malaysia DigitalDome Photography Terengganu Malaysia

**Keywords:** *
Thismia
*, taxonomy, mycoheterotrophy, new species, Telemong Forest Reserve, Terengganu, Peninsular Malaysia

## Abstract

Two new species of the mycoheterotrophic genus *Thismia* Griff. (Thismiaceae), *Thismiadomei* Siti-Munirah and *T.terengganuensis* Siti-Munirah from Peninsular Malaysia, are described and illustrated. *Thismiadomei*, characterized by its perianth lobes that are upright and curve inward, but are imperfectly connate, falls within sectionOdoardoa. *Thismiaterengganuensis* is characterized by its mitre with three appendages on its apex, so falls within sectionGeomitra. Both new species are unique and totally different from other described species, *T.domei* by the trichomes on its outer perianth tube surface and *T.terengganuensis* by its mitre with slender appendages. *Thismiajavanica* J.J.Sm, also from Terengganu, is a new record for Peninsular Malaysia.

## Introduction

*Thismia*, a genus of small, mycoheterotrophic herbs, currently comprises about 70 accepted species (Hroneš et al. 2018; Suetsugu et al. 2018). In 2018, eight new species were described from East Malaysia (Sabah and Sarawak). We can expect more new species in future. *Thismia* is poorly known because the above-ground parts are ephemeral and are often overlooked due to their small size. Well-preserved herbarium specimens are rare but spirit-preserved material and field images are informative. Currently in Malaysia, including these two new species and the new record, there are 27 species. Thirteen occur in Peninsular Malaysia, namely: *Thismiaalba* Holttum *ex* Jonker, *T.arachnites* Ridl., *T.aseroe* Becc., *T.chrysops* Ridl., *T.clavigera* F. Muell., *T.crocea* (Becc.) J.J.Sm, *T.fumida* Ridl., *T.grandiflora* Ridl., *T.javanica* J.J. Sm., *T.kelantanensis* Siti-Munirah, *T.racemosa* Ridl., (Jonker 1948, Siti Munirah 2018) and the two new species described here. However, based on Jonker (1948), *T.crocea* is considered as highly dubious for Peninsular Malaysia. Its status in Peninsular Malaysia needs to be revisited.

Endemism is high, about half the Peninsular Malaysian species are endemic to Peninsular Malaysia and *T.kelantanensis*, *T.racemosa*, *T.grandiflora* and *T.chrysops* have only been collected once. *Thismiaalba*, *T.arachnites*, *T.clavigera* and *T.javanica* also occur in Thailand; and *T.fumida* and *T.aseroe* in Singapore. The most common species, which is found from many places in Peninsular Malaysia, is the bright yellow species, *T.alba*.

In 2018, six new species were described from Sarawak and two from Sabah. Sochor et al. (2018a) recently rediscovered the magnificent *Thismianeptunis* in Sarawak after 150 years. In total, Sarawak has about 10 species of which 9 are endemic: *Thismiaacuminata* Hroneš, Dančák & Sochor, *T.laevis* Sochor, Dančák & Hroneš, *T.nigra* Dančák, Hroneš & Sochor, *T.viridistriata* Sochor, Hroneš & Dančák, (Sochor et al. 2018b), *Thismiacornuta* Hroneš, Sochor & Dančák (Hroneš et al. 2018), *T.kelabitiana* Dančák, Hroneš & Sochor (Dančák et al. 2018), *T.bifida* M.Hotta, *T.clavigera*, *T.episcopalis* (Becc) F.Muell, *T.neptunis* Becc. (Jonker 1948). Five taxa have been described from Sabah of which three are endemic: *Thismiabryndonii* T*s*ukaya, Suetsugu & Suleiman (Tsukaya et al. 2017), T.hexagonavar.grandiflora (Tsukaya et al. 2014), *T.goodii* Kiew (Kiew 1999), *T.kinabaluensis* Nishioka & Suetsugu (Nishioka et al. 2018) and *T.pallida* Hroneš, Dančák & Rejžek (Hroneš et al. 2018).

**Map 1.**
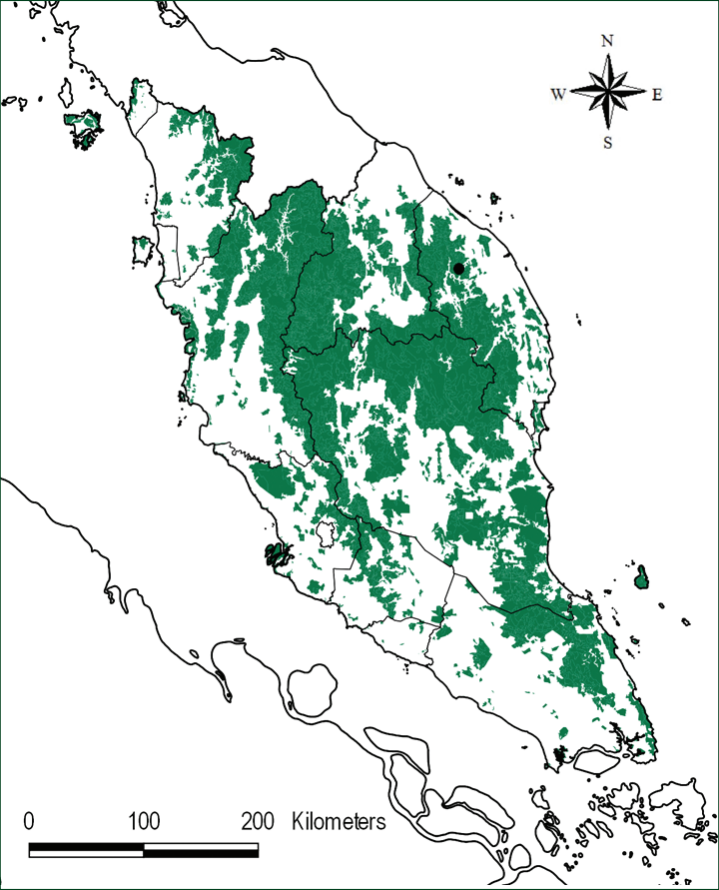
Hulu Telemong Forest Reserve (●), the type locality of *Thismiadomei* and *T.terengganuensis* and the locality of *T.javanica*. NFI III Courtesy of Forest Department Peninsular Malaysia.

The two new species and *Thismiajavanica* were discovered by the second author, Mr Dome Nikong during a photographic trip in the Hulu Telemong Forest Reserve (FR) area, in the state of Terengganu, Peninsular Malaysia (Map 1). Hulu Telemong FR, located to the north of Kenyir Lake, was previously logged for timber. However, some patches of pristine primary rain forest have survived.

## Materials and methods

This study is based on material collected by the second author in November and December 2018 from Hulu Telemong FR, Kuala Berang District, Terengganu. The specimens were preserved in spirit. Morphological characters were studied using stereo microscope and high-resolution macrophotography. Measurements were taken from live and spirit material. The specimen details were compared in detail with original drawings and descriptions given in the protologues of *Thismia* species in the Malaysian region.

## Taxonomic account

### Thismia domei

Taxon classificationPlantaeDioscorealesBurmanniaceae

M.Y.Siti-Munirah sp. nov.

urn:lsid:ipni.org:names:77198712-1

Figures 1 , 2 , 3

#### Diagnosis.

The whole plant is completely white translucent and the flower is strigose with white trichomes covering the outer surface of the perianth tube and ovary; the apex of the perianth tube is partially covered by the apical part of flower tube with a ring-like annulus and together with six perianth lobes are upright and curve inwards with a dorsal long appendage on each lobe.

#### Type.

MALAYSIA. Peninsular Malaysia: Terengganu, Kuala Berang District, Hulu Telemong Forest Reserve, ca 207 m alt., 22 Nov 2018, *Dome Nikong, FRI 91111* (holotype KEP!).

#### Description.

Terrestrial, achlorophyllous, whitish herb to 9 cm tall. Roots vermiform brownish with white apices. Stems erect (sometimes curved), unbranched, 0.2 to 2.5 cm long, glabrous with ridges. Leaves scale-like, appressed, 2–7 mm long, 2 mm wide, smaller at the base leaf increasing in size above, alternate, single, triangular to lanceolate, translucent white, apex acute or acuminate, base appressed. Internodes 4–10 mm. Involucral bracts 3, translucent white, ca. 1–1.2 cm long, lanceolate, apex acute to acuminate, margin entire, base appressed, with a central vein. Bud to 7 mm long, pale to dark purple. Pedicel 4–5 mm long. Flowers solitary to 7–8 cm long (including appendages); perianth lobes 6, each 3 × 2 mm (excluding appendage), obovate to spathulate are upright and curve inwards over perianth chamber, apex truncate, with erect subulate appendage abaxially, ca. 3.5–5.5 cm long, ca. 0.5 mm wide, cylindric, apex rounded or falcate; perianth tube bowl-like, 5–5.5 mm long, 3–6 mm wide, narrowed just above the ovary, widest just below the lobes, white translucent, outer surface with longitudinal ribs covered with numerous white translucent thick trichomes; apical part of perianth tube with annulus and opening circular aperture ca. 2 mm in diameter, from above ring-like, whitish, glabrous, Stamens 6, pendulous attached to the inner wall of perianth tube, ca. 2.5 mm long, apex lobed with 3 apical appendages with glandular tip (visible in fresh plant); filaments short, free, white, each with two rounded oblong thecae facing the inner wall of the perianth tube; anthers oblong, 2 mm long; lateral appendage box-shaped; ovary inferior, cup-shaped, ca. 4 mm long, whitish to brownish, outer surface smooth, without longitudinal ribs, covered with numerous white translucent thick trichomes, unilocular with 3 parietal placentas; style ca. 0.5 mm long; stigmas 3, ca. 0.6 mm long, oblong, papillate, 3-lobed, lobes slightly folded, apex truncate. Fruit cup-shaped, translucent white.

#### Distribution.

Endemic in Peninsular Malaysia, Terengganu. Currently known only from the type collection.

#### Ecology.

In lowland dipterocarp forest on wet, moist soil in shade at an altitude of 207 m. Flowering in November-December. This new species was encountered on bamboo leaf litter near an elephant trail. When mature *T.domei* produces an unpleasant smell like rotting fish.

#### Etymology.

The species is named after Mr Dome Nikong, professional photographer and freelance researcher, who first discovered this species and the other new species described below as well as *T.javanica*.

#### Conservation status.

Critically Endangered (B2 ab(iii)). Following the 2012 IUCN Red List Categories and Criteria, (IUCN 2012), this species is assessed as critically endangered because it is only known from the type locality. It is very rare. Fewer than 10 individuals were observed, which included both flowering and fruiting individuals. The type locality is within a forest reserve in disturbed forest area near the river bank at an elevation of c. 207 m. The species is under threat because selective logging activities are currently on-going within the forest reserve.

#### Notes.

*Thismiadomei*, based on colour, is most like *T.clavarioides* K.Thiele (Thiele and Jordan 2002) and *Thismiataiwanensis* S.-Z. Yang, R. M. K. Saunders & C.-J. Hsu, (Yang et al. 2002). Both have completely white perianth lobes that are upright and curved inwards towards the perianth tube with an appendage on each lobe. However, *T.domei* differs from these species in that its perianth tube is fully covered with thick trichomes and it has six perianth lobes each with an appendage as opposed to being completely glabrous and *T.taiwanensis* from Taiwan has only three perianth lobes with appendages and the perianth of *T.clavarioides* forms a mitre from Australia. *T.domei* is unusual in its conspicuous cell inclusion white spot which appear to be aleurone grains.

**Figure 1.**
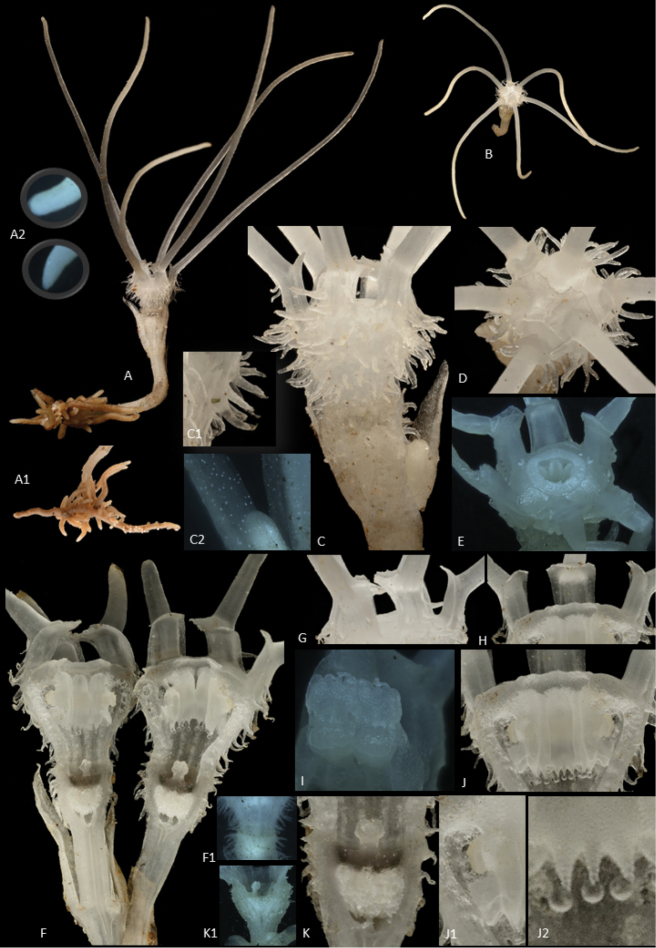
*Thismiadomei* Siti-Munirah **A** habit (**A1** roots **A2** tip of filiform appendages), **B** top view of plant **C** perianth tube covered with trichomes (**C1** trichome **C2** white spots **D** top view of flower showing perianth lobes overlapping and appendages on the each lobes forming loose mitre **E** top perianth tube covered with apical part of flower tube and annulus **F** longitudinal section of flower (**F1** base perianth tube and ovary) **G** perianth lobes from outside **H** perianth lobes from inside **I** pendulous stamen attached to the inner wall of perianth tube (abaxial view of stamens) **J** pendulous stamen attached to the inner wall of perianth tube (adaxial view of stamens) (**J1** stamen from side view **J2** three appendages at apical margin) **K** ovary showing stigma (**K1** ovary). Photo credit: **A**, **A1**, **B**, **C**, **C1**, **D**, **F**, **G**, **H**, **J**, **J1**, **J2**, **K** Dome Nikong; **A2**, **C2**, **E**, **F1**, **I**, **K1** Siti-Munirah MY.

**Figure 2.**
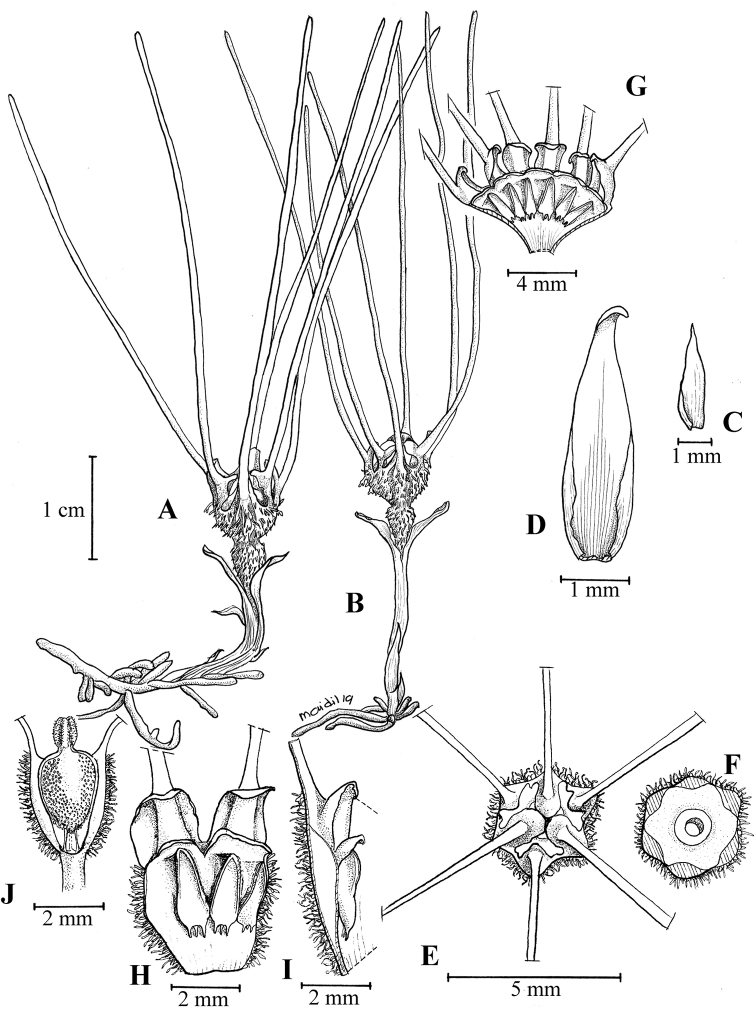
*Thismiadomei* Siti-Munirah **A, B** habit of two plants **C** leaf **D** bract **E** perianth lobes from top **F** top view of apical part of perianth tube with annulus **G** inner adaxial view of six pendulous stamens **H** stamen attached to perianth tube **I** a perianth lobe with perianth tube from side view **J** ovary with stigma and placenta (below). All from Dome Nikong FRI 91111. Drawn by M. Aidil. The drawing is based on spirit material.

**Figure 3.**
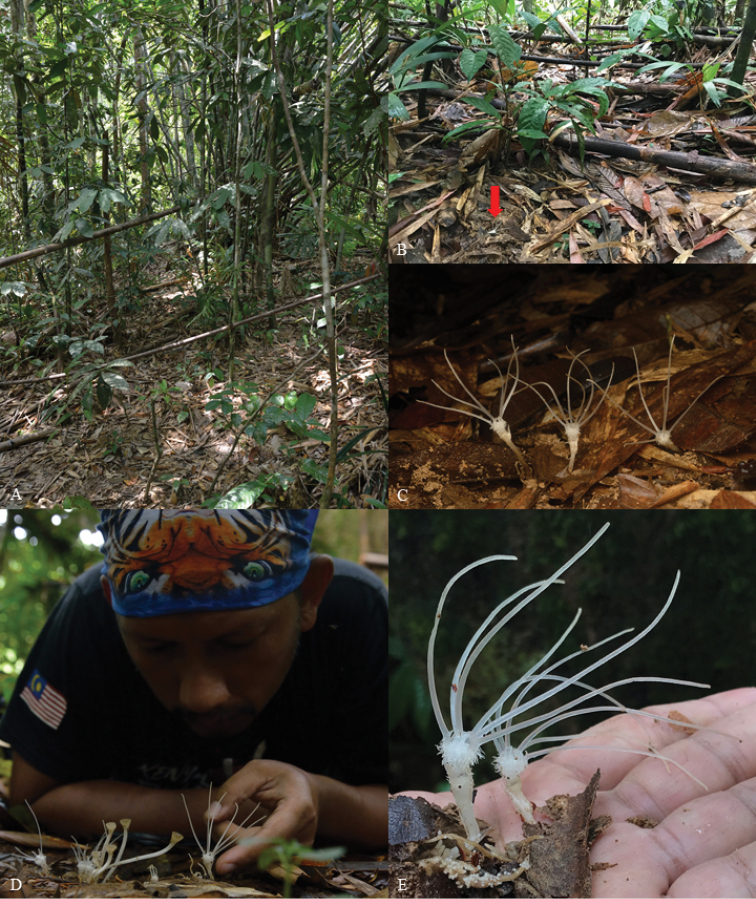
*Thismiadomei*. **A, B** habitat on leaf litter within a bamboo clump (plant indicated by red arrow) **C** habit of the flowering *T.domei***D** Mr Dome Nikong showing *T.domei* at different stages of anthesis **E** habit of *T.domei*. Photo credit: Dome Nikong.

### Thismia terengganuensis

Taxon classificationPlantaeDioscorealesBurmanniaceae

M.Y.Siti-Munirah sp. nov.

urn:lsid:ipni.org:names:77198713-1

Figure 4 , 5 , 6

#### Diagnosis.

*Thismiaterengganuensis* is unique in its perfect mitre with the long filiform appendages not seen in any other species of *Thismia*. It has a racemose sessile inflorescence with large bracts. Its flower parts are brownish with brown striae with inner perianth lobes forming a dark brown mitre with a white slender appendage attached at apex. The outer perianth lobes at interval appear like a wing. It has creeping vermiform brown roots.

#### Type.

MALAYSIA. Peninsular Malaysia: Terengganu, Kuala Berang District, Hulu Telemong Forest Reserve, ca 227 m alt., 2 Dec 2018, *Dome Nikong, FRI 91112* (holotype KEP!).

#### Description.

Small perennial achlorophyllous herbs. Roots creeping, vermiform, brown. Stem white, 3–3.5 cm long, ascending, glabrous. Leaves scale-like, triangular, ca. 3 mm long, apex acute, alternate. Bracts many, large, 1–1.5 cm long, crowded. Pedicel short or up to 3–4 cm long. Inflorescence racemose. Flowers sessile, perianth tube urceolate, ca. 1 cm long, 5 mm wide in the upper third, brownish-white, with inconspicuous brown longitudinal stripes from upper part to the base. Apex of perianth tube covered with the dark brown apical part of perianth tube, circular, slightly raised annulus with opening aperture ca. 2 mm in diameter. Perianth lobes divided into 2 types; inner perianth lobes 3, erect, convergent to connate at apex, forming a mitre, dark brown, each with long and slender filiform appendages, 5–7 cm long; outer perianth lobes 3, recurved, all equal in shape and size, long triangular, boat-like, 4–5 mm long, 2 mm wide at base, white, tapering into curved apex. Stamens 6, brownish white; filaments short, free, attached to the mouth of the perianth tube, curved downwards; connectives blunt (tongue-like), without apical appendages; thecae yellow, not connected; lateral appendage skirt-like. Ovary white, cup-shaped, inferior, free central placentation; style short; stigma 3-lobed, papillose. Fruit cup-shaped, brownish.

#### Distribution.

Endemic in Peninsular Malaysia, Terengganu. Currently only known from the type locality.

#### Ecology.

In lowland dipterocarp forest on wet, moist soil in shade at altitude 227 m. Flowering in November-December on forest floor under canopy of dense shrubs.

#### Etymology.

The epithet refers to the state, Terengganu, where it was found.

#### Conservation status.

Critically Endangered (B2 ab(iii)). Following the 2012 IUCN Red List Categories and Criteria, (IUCN 2012), this species is assessed as critically endangered because it is only known from one locality where less than 5 individuals flowering and fruiting individuals were observed. *Thismiaterengganuensis* is currently known only from the type locality and is certainly a very rare species. The locality is within the forest reserve but is threatened by selective logging activities within the forest reserve that are currently ongoing.

#### Notes.

*Thismiaterengganuensis* is most similar to species in sect.Sarcosiphon and sect.Geomitra in the shape of the perianth tube and mitre but differs in all other morphological parts, e.g. in its slender filiform appendages on the apex of mitre and also its connectives that are blunt without any apical appendages.

**Figure 4.**
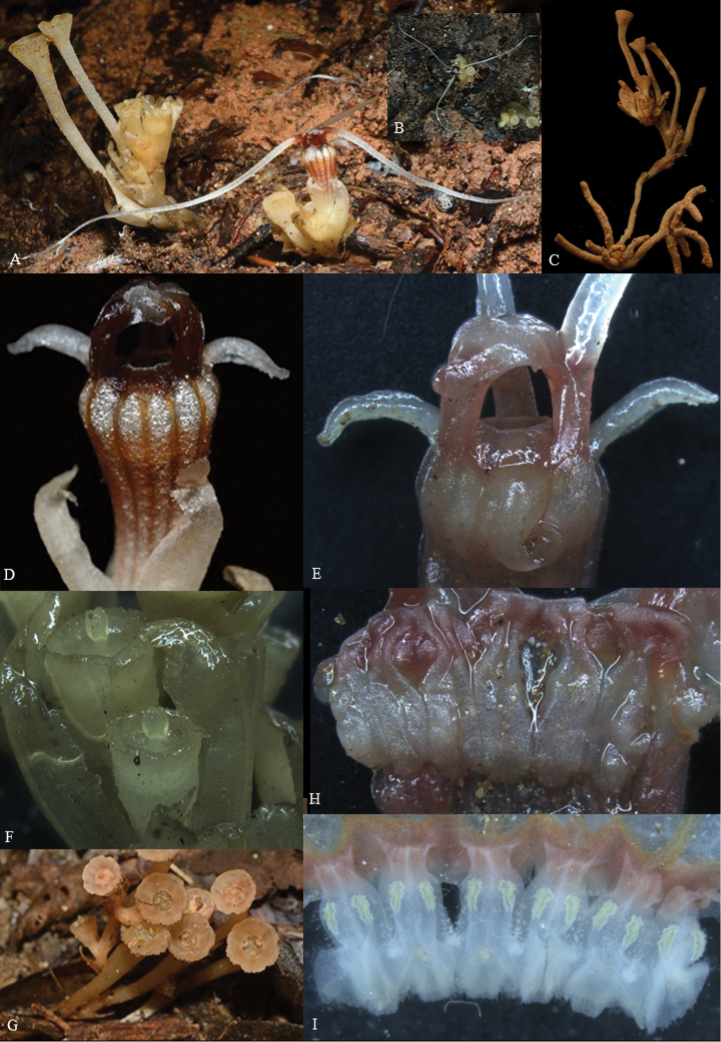
*Thismiaterengganuensis* Siti-Munirah **A** habit of flowering and fruiting plants **B** top view of plant **C** root and stems **D** perianth tube **E** perianth lobes (inner forming a mitre, outer curved) **F** ovary with pistil (stigma) **G** fruits with seeds **H** stamens attached to the inner wall of mouth of the perianth tube (adaxial view) **I** stamens deflexed (abaxial view). Photo credit: **A–D**, **G** Dome Nikong; **E**, **F**, **H**, **I** Siti-Munirah MY.

**Figure 5.**
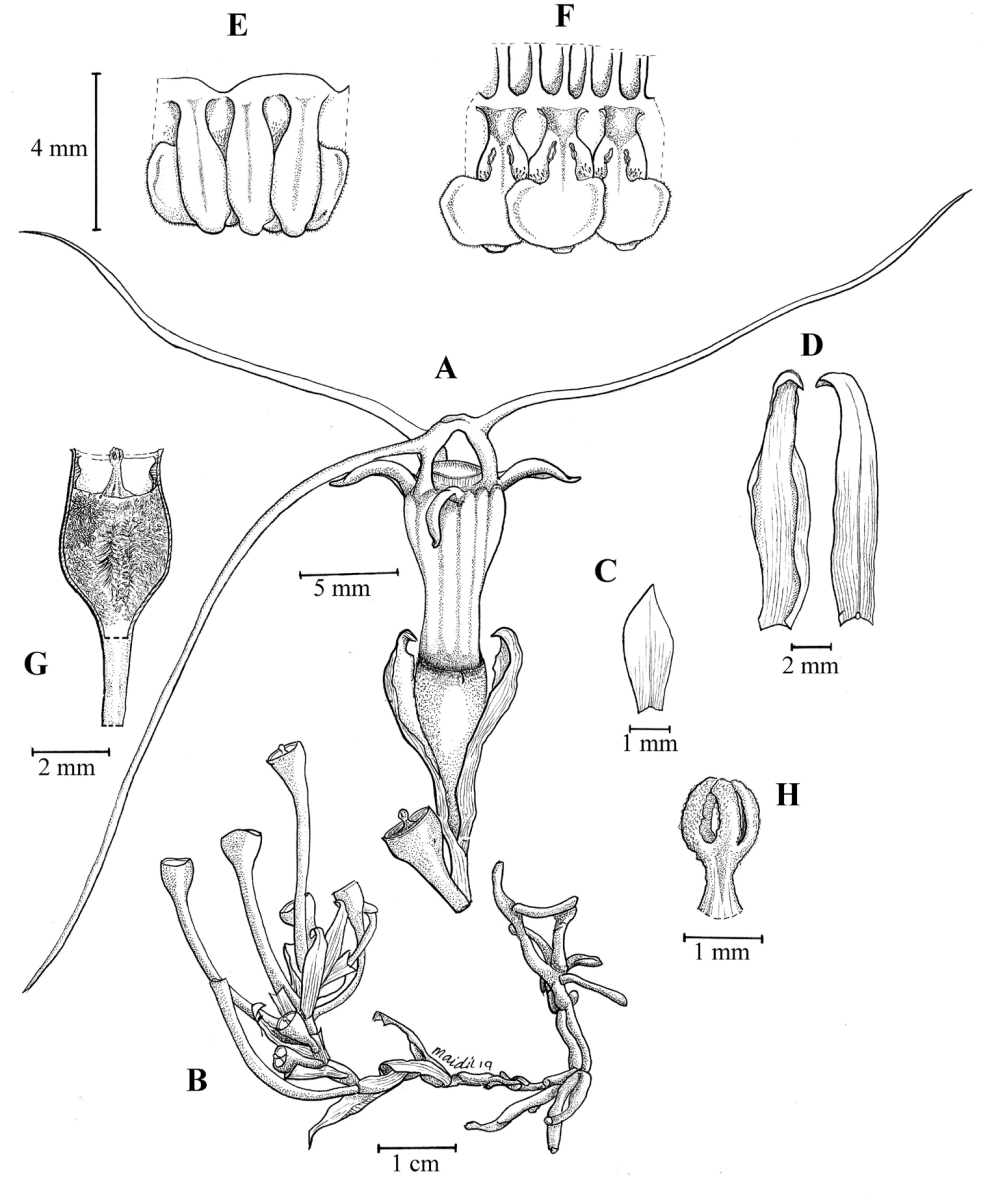
*Thismiaterengganuensis* Siti-Munirah **A** flower and fruit **B** habit of fruiting plant **C** leaf **D** bracts **E** stamens (adaxial view) **F** stamens (abaxial view) **G** ovary showing free central placentation **H** stigma. All from Dome Nikong FRI 91112. Drawn by M. Aidil.

**Figure 6.**
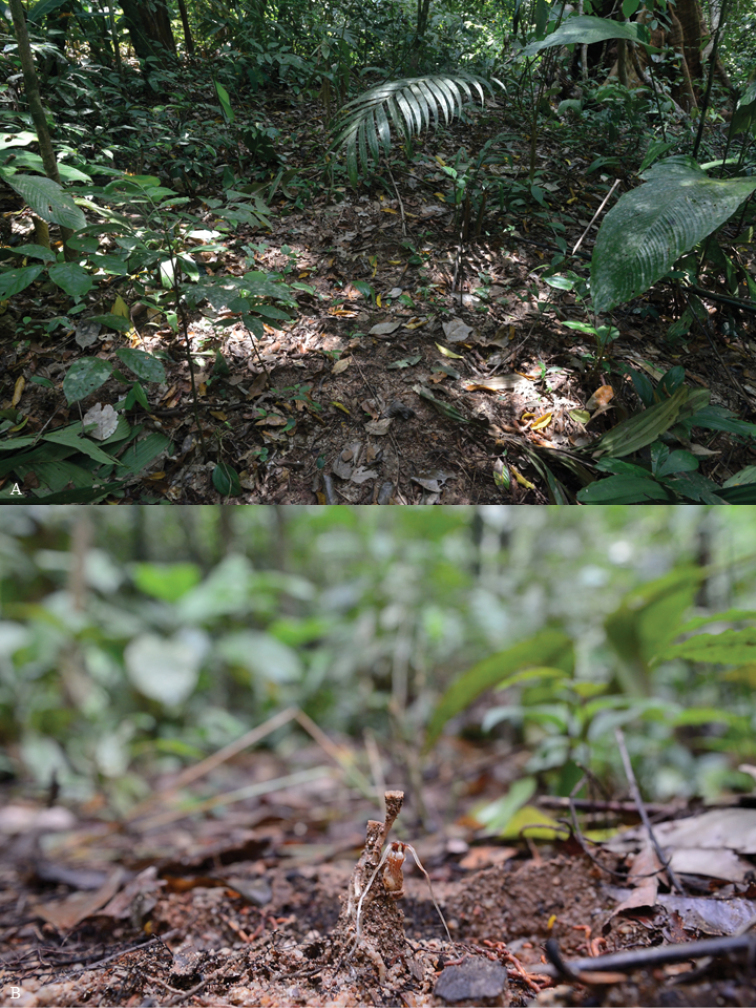
*Thismiaterengganuensis***A** habitat **B** habit of a fading *T.terengganuensis* plant. Photo credit: Dome Nikong.

### New record

#### Thismia javanica

Taxon classificationPlantaeDioscorealesBurmanniaceae

J.J. Sm., Ann. Jard. Bot. Btzg. 23: 32. 1910

Figure 7

Thismia javanica J.J. Sm., Ann. Jard. Bot. Btzg. 23: 32. 1910; Jonker, Fl. Malesiana 1,4: 23. 1948; Larsen, Fl. Thailand 5,1: 125. 1987. Specimen: *Dome Nikong FRI 91114* (KEP!)

##### Note.

Stem erect simple, rarely branched, up to 12 cm tall, 1-few flowered. Leaves scale-like, lanceolate to ovate, 3 mm long. Involucral bracts 3, orange. Perianth-tube urceolate, pale orange with darker stripes, with longitudinal bars inside connected by several transverse bars; outer perianth lobes orange, ovate, inner ones triangular, terminated by a 2-3 cm long appendage. Anthers 3-toothed at the apical margin, each tooth terminated by a hair; connective broad with quadrangular appendage. Ovary obovoid; style short; stigma truncate. Fruit orange, 6 mm long.

##### Distribution.

Indonesia, Malaysia and Thailand.

##### Conservation status.

We propose a regional conservation status for *T.javanica* in Peninsular Malaysia as Critically Endangered (B2 ab(iii)). Following the 2012 IUCN Red List Categories and Criteria, (IUCN 2012), this species is assessed as critically endangered because it is only known from one locality where less than 6 individuals flowering and fruiting individuals were observed. It lies within a forest reserve that is threatened by selective logging activities that are currently on-going.

##### Notes.

The specimens of *Thismiajavanica* were found not far from the *T.terengganuensis* population. We believe that *T.javanica* has a wider distribution in Peninsular Malaysia based on photographs of a specimen from Langkawi, Kedah, by late Abd Ghani Hussain. Unfortunately, there are no specimen to verify this.

##### Discussion.

The genus Thismiais divided into two subgenera,subg.Ophiomeris (Miers) Maas & Maas and subg.Thismia (Kumar et al., 2017). All Peninsular Malaysian species belong to subgenusThismia. It is divided into five sections of which three occur in Peninsular Malaysia, (a) sect. Thismiawith two subsections,subsect.Brunonithismia Jonker (*T.arachnites*, *T.javanica*) and subsect.Odoardoa Schlechter (*T.alba*, *T.aseroe*, *T.chrysops*, *T.domei*, *T.fumida*, *T.grandiflora*, *T.racemosa*), (b) sect.Sarcosiphon (Blume) Jonker (*T.crocea*), and (c) sect.Geomitra Kumar & S.W. Gale (*T.kelantanensis*, *T.clavigera* and *T.terengganuensis*). *Thismiaterengganuensis* is a unique species which can hardly be assigned to any section of *Thismia*. However, we currently locate it under sect.Geomitra as its perianth lobes form a mitre with appendages.

**Figure 7.**
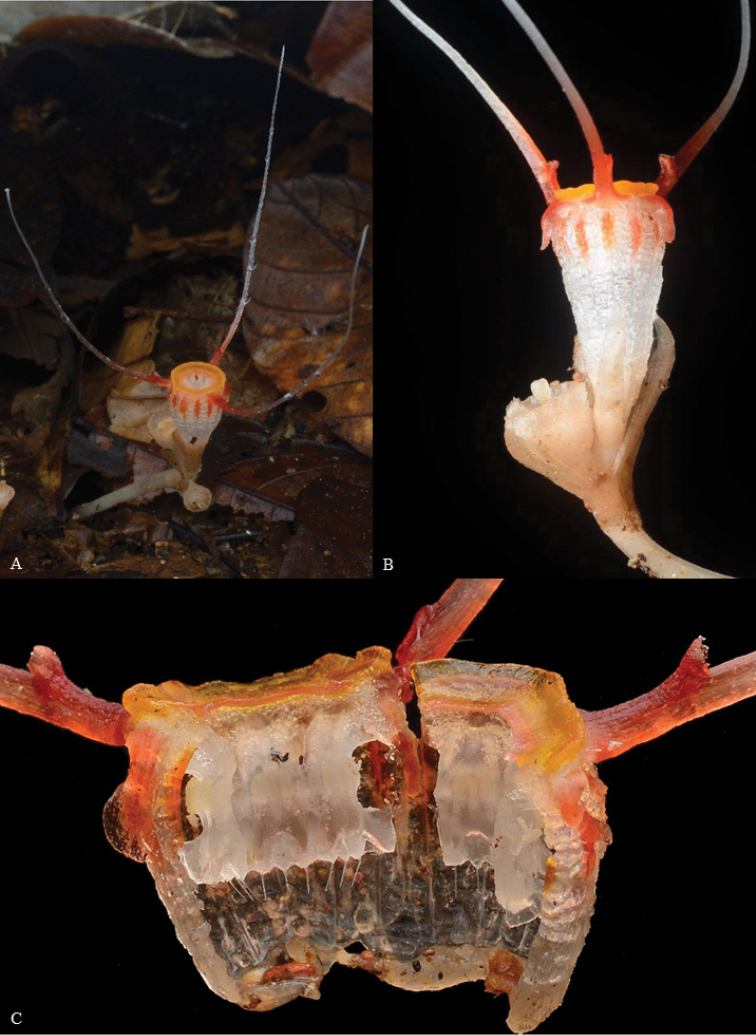
*Thismiajavanica* from Terengganu **A** habit **B** flower **C** opened to show the inside of the perianth tube. Photo credit: Dome Nikong.

## Supplementary Material

XML Treatment for Thismia domei

XML Treatment for Thismia terengganuensis

XML Treatment for Thismia javanica
